# Effectiveness of Treatment Approaches for Children and Adolescents with Reading Disabilities: A Meta-Analysis of Randomized Controlled Trials

**DOI:** 10.1371/journal.pone.0089900

**Published:** 2014-02-26

**Authors:** Katharina Galuschka, Elena Ise, Kathrin Krick, Gerd Schulte-Körne

**Affiliations:** 1 Department of Child and Adolescent Psychiatry and Psychotherapy, University of Munich, Munich, Germany; 2 Department of Child and Adolescent Psychiatry and Psychotherapy, University of Cologne, Cologne, Germany; University Children’s Hospital Tuebingen, Germany

## Abstract

Children and adolescents with reading disabilities experience a significant impairment in the acquisition of reading and spelling skills. Given the emotional and academic consequences for children with persistent reading disorders, evidence-based interventions are critically needed. The present meta-analysis extracts the results of all available randomized controlled trials. The aims were to determine the effectiveness of different treatment approaches and the impact of various factors on the efficacy of interventions. The literature search for published randomized-controlled trials comprised an electronic search in the databases ERIC, PsycINFO, PubMed, and Cochrane, and an examination of bibliographical references. To check for unpublished trials, we searched the websites clinicaltrials.com and ProQuest, and contacted experts in the field. Twenty-two randomized controlled trials with a total of 49 comparisons of experimental and control groups could be included. The comparisons evaluated five reading fluency trainings, three phonemic awareness instructions, three reading comprehension trainings, 29 phonics instructions, three auditory trainings, two medical treatments, and four interventions with coloured overlays or lenses. One trial evaluated the effectiveness of sunflower therapy and another investigated the effectiveness of motor exercises. The results revealed that phonics instruction is not only the most frequently investigated treatment approach, but also the only approach whose efficacy on reading and spelling performance in children and adolescents with reading disabilities is statistically confirmed. The mean effect sizes of the remaining treatment approaches did not reach statistical significance. The present meta-analysis demonstrates that severe reading and spelling difficulties can be ameliorated with appropriate treatment. In order to be better able to provide evidence-based interventions to children and adolescent with reading disabilities, research should intensify the application of blinded randomized controlled trials.

## Introduction

Children, adolescents, and adults with reading disability (dyslexia) experience a significant impairment in the acquisition of reading accuracy, reading fluency, reading comprehension, and spelling skills, which cannot be accounted for by low IQ, visual acuity problems, neurological damage, or poor educational opportunities [1]. Reading disability has genetic basis [2] and the underlying neurobiological and cognitive causes are largely debated. Impairments in auditory speech perception and processing, as well as visual attention and perception deficits are considered as the main causes of reading and spelling difficulties in dyslexia [3]–[5]. Reading and spelling deficits influence an individual’s performance in most academic domains [1]. In addition, there is strong evidence of a link between reading disabilities and externalizing disorders, generalized anxiety, and school-related anxiety [6], [7].

The evidence-based development and the evaluation of interventions for children and adolescents with reading disabilities are, therefore, of particularly profound importance. A large number of interventions and therapies, derived from various treatment approaches, have been constructed and evaluated. Several systematic reviews have already summarized the findings of studies that evaluated the effectiveness of reading and spelling interventions. One of the most influential reviews of the research literature was conducted by the National Reading Panel (NRP) in the year 2000 [8]. The review displays important results about the effectiveness of different types of reading instruction. Its main finding was that systematic instruction in learning letter sound relations and in blending sounds to form words is most effective for improving reading and spelling skills in disabled readers [8]. Despite the importance of this finding, 13 years after its publication, the NRP review needs to be updated in order to integrate recent empirical findings.

However, most current systematic reviews are focused on the effectiveness of one specific treatment approach [9]–[11]. Other reviews address preventive methods for children at-risk for reading disability [12], [13]. Since there is no widespread use of randomized-controlled trials (RCTs) in this research domain, current systematic reviews and meta-analyses often included not only RCTs, but also low-quality primary research (e.g., non-randomized research designs) [14]–[16]. To our best knowledge, no systematic review has been published to date that includes all available RCTs, without focusing on a specific treatment approach, and that integrates the results quantitatively with statistical methods.

The present meta-analysis has two advantages over previously published work. First, due to the inclusion of exclusively RCTs, the observed effect sizes can most likely be attributed to the intervention. Second, because all available RCTs are integrated, it is possible to compare the effectiveness of different treatment approaches.

The goal of this meta-analysis is twofold. The first aim is to determine the efficacy of different treatment approaches on reading and spelling performance of reading disabled children and adolescents. The second aim is to explore the impact of various factors on the efficacy of these treatment approaches.

## Methods

### Literature Search

An extensive literature search was conducted. We searched for intervention studies that were published until June 2013 in the databases ERIC, PsycINFO, PubMed, and Cochrane with the following search terms: “dyslexia” or “developmental reading disorder” or “developmental dyslexia” or “developmental reading disability” or “reading disorder” or “word blindness” or “spelling disorder” or “developmental spelling disorder” or “specific spelling disorder” combined with “intervention” or “treatment” or “therapy” or “therapeutics” or “training” or “remediation”.

We also examined bibliographical references of systematic reviews and primary studies. To check for unpublished RCTs, we searched the websites clinicaltrials.com and ProQuest. In addition, we contacted experts by sending an e-mail to each member of the mailing list of the Society for the Scientific Studies of Reading (SSSR).

### Study Selection Criteria

**Figure 1:**
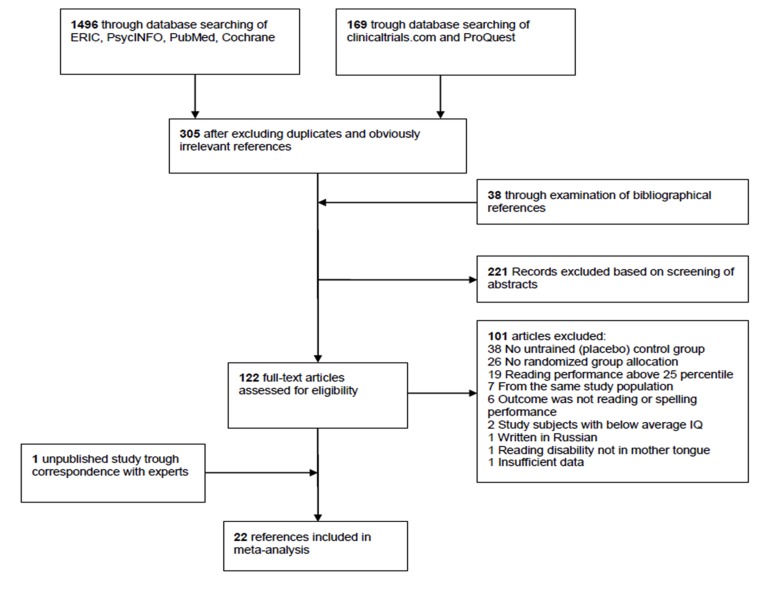
Flow chart of study selection process.

To be considered for this review, studies must have met the following criteria: (a) the aim of the study was to examine the efficacy of an intervention or remediation programme for children and adolescents with reading disabilities; (b) the manuscript was written in English; (c) the study design included an untrained control group or a placebo training group; (d) group allocation was randomized, including parallel group randomization, group cluster randomization (quasi-randomized controlled trials were not selected); (e) study subjects were children, adolescents or adults (no studies with adults could be included) whose reading performance was below the 25^th^ percentile or at least one standard deviation (SD), one year, or one grade below the expected level; (f) the study included subjects with intelligence in the normal range (IQ≥85, or described as having normal intelligence by the study author); (g) poor reading occured in mother tongue; (h) one or more reading or spelling tests were administered before and after treatment; and (i) pre- and post-test results of the reading or spelling tests were reported with sufficient detail to allow the calculation of an effect size or could be requested from the authors. Figure 1 summarizes the process of selecting studies for the meta-analysis.

### Coding of the RCTs

Coding was done independently by the first author and an associate using a structured coding sheet. First, data necessary for effect size calculation (mostly means and standard deviations of pre- and post-tests) was extracted. Next, methodological characteristics, intervention characteristics, and sample characteristics were coded.

The methodological characteristics included: (a) the dependent variable (reading speed, reading comprehension, reading accuracy, pseudoword reading accuracy, pseudoword reading speed, nonword reading accuracy, nonword reading speed, or spelling); (b) the sample size; and (c) the administered reading test and spelling test. The intervention characteristics included: (a) treatment approach; (b) spelling/writing activities included (yes or no); (c) duration of the intervention in weeks; (d) total amount of intervention in hours; (e) setting (group, individual, or computer); and (f) conductor (professional or nonprofessional).

Treatment approaches were classified into distinct categories based on the description of the intervention in the report. The categories closely match the topic areas of the NRP review [8]. The category *phonemic awareness instruction* includes interventions that foster the ability to recognize and manipulate phonemes in words. This implies tasks for recognizing phonemes in words, blending phonemes to words, segmenting a word into its phonemes, eliminating a phoneme from a word, or adding a phoneme to a word. All tasks are presented and performed orally. The category *phonics instruction* includes interventions that systematically teach letter-sound-correspondences and decoding strategies that involve blending or segmenting individual letters or phonemes or dividing a spoken or written word into syllables or onset and rimes. These interventions comprise reading and writing activities. The category *reading fluency training* includes interventions that contain repeated oral word reading practice or guided repeated word reading. These interventions aim to improve word recognition skills. The category *reading comprehension training* includes interventions that comprise tasks in which participants learn to extract textual information, summarize it, and relate it to existing knowledge. The category *auditory training* includes interventions in which subjects are confronted with non-linguistic auditory stimuli and are trained to identify and distinguish these stimuli. The category *medical treatment* includes interventions in which participants receive drugs to enhance their reading and spelling performance. The category *coloured overlays* includes interventions in which study subjects read with coloured filters or coloured overlays.

Finally, sample characteristics were coded. These included (a) age (mean and range) and (b) severity of reading impairment. The severity of reading impairment was identified by the inclusion criteria used in the trials and consists of three categories. The category *severe reading disability* includes studies in which participants’ reading performance was at least 2 SD below the expected value, below the 2.5^th^ percentile, at least two years below grade level, or showed a discrepancy between chronological age and reading age of at least two years. The category *moderate reading disability* includes studies in which participants’ reading performance was at least 1 SD below the expected value, at least one year below grade level, below the 16^th^ percentile, or showed a discrepancy between chronological age and reading age of at least one year. The category *mild reading disability* includes studies in which participants’ reading performance was below the 25^th^ percentile.

### Data Extraction and Effect Size Calculation

To evaluate the efficacy of an intervention, the effect size Hedges *g* was calculated by dividing the difference between the performance scores of the control group (CG) and the experimental group (EG) at post-test by their pooled standard deviation, and multiplying the result by a correction factor [17], [18].

Formula 1 - Hedges *g*


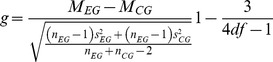


M = mean; EG = experimental group; CG = control group; n = number of study subjects; s = standard deviation; df = degrees of freedom.

If studies included more than one intervention group, but only one control group, every comparison of an intervention group with the control group was treated separately as an individual study. As a consequence, the control group was used to compute several effect sizes which are not independent from each other. An overweighting of the effect sizes was counteracted by dividing the sample size of the control group by the number of intervention groups. Similarly, if several control groups, but only one intervention group, were included, each comparison of a control group with the intervention group was treated as an individual study and the sample size of the intervention group was divided by the number of control groups.

To reduce the risk of under- or overestimating effect sizes, some effect sizes were corrected for pre-test differences. If the difference between the pre-test scores of the experimental and the control group displayed an effect size equal or greater than 0.20 (*g*≥0.20), the post-test score of the experimental group was corrected by adding or subtracting the difference between the pre-test scores. The effect size was then calculated on the basis of the corrected post-test score and the (uncorrected) pooled standard deviation. This was done because the formula described above does not take into consideration the pre-test differences, which leads to an over- or underestimation of the true magnitude of the effect if there are significant differences between the groups before the start of the intervention.

A maximum of two effect sizes were calculated for each comparison of an experimental group with a control group, one for reading performance and one for spelling performance. The following measures of reading performance were considered adequate for effect size calculation: reading accuracy, reading speed, reading comprehension, nonword reading speed, nonword reading accuracy, pseudoword reading speed or pseudoword reading accuracy. To determine spelling performance, tests measuring spelling accuracy were considered adequate.

Some studies used multiple reading and spelling tests to determine treatment efficacy, including standardized measures and non-standardized measures of learning transfer, as well as non-standardized measures whose tasks closely matched the training content. Effect sizes were calculated based on standardized measures, which are generally considered to be measures of learning transfer, if these were available. If standardized measures were not available, non-standardized measures of learning transfer were used for effect size calculation (*n* = 3 studies). Self-constructed measures that matched the training content were not used for effect size calculation, because these measures may not generalize to material not specifically taught. Thus, all effect sizes are based on measures of learning transfer. If a study reported results for several comparable tests (e.g., several standardized tests measuring different aspects of reading such as reading speed and comprehension), an average effect size was calculated from the effect sizes for individual tests, separately for reading and spelling performance.

Non-standardized dependent measures are suspected to overestimate the true magnitude of an effect [14], [19]. Although all effect sizes are based on measures of learning transfer, it cannot be ruled out completely that the inclusion of studies without standardized measures introduced an artifact. For this reason, the main analyses were run with and without studies that used non-standardized measures. First, the analyses were conducted with all studies that met the inclusion criteria outlined above (i.e. studies with standardized or non-standardized measures; *n* = 22 studies; see Table 1). Second, the analyses were run with those studies that included standardized measures (*n* = 19 studies).

**Table 1:** Efficacy of treatment approaches on reading performance.

				95% CI		Heterogeneity				Significance		
Variable	Value	N	*g’*	Lower	Upper	*Q*	df	*p*	*I^2^*	*Q*	df	*p*
Treatment approach	Phonemic awareness instruction	3	0.279	−0.244	0.802	3.663	2	0.160	45%	3.164	6	0.788
	Phonics instruction	29	0.322	0.177	0.467	26.810	28	0.529	0%			
	Reading fluency training	5	0.301	−0.105	0.707	1.389	4	0.845	0%			
	Reading comprehension training	3	0.177	−0.181	0.535	0.525	2	0.769	0%			
	Auditory training	3	0.387	−0.065	0.838	2.053	2	0.358	3%			
	Medical treatment	2	0.125	−0.072	0.322	1.331	1	0.249	25%			
	Coloured overlays	4	0.316	−0.012	0.644	0.885	3	0.829	0%			

For studies that did not report means and standard deviations, effect sizes were calculated on the basis of other measures, for example t-test or F-test values. If a study did not report sufficient data, more information was requested from the corresponding author. If this request failed, co-authors were contacted.

### Quality Assessment

The methodological quality of the included studies was assessed independently by the first author and an associate with the checklist for randomized controlled trials by the Scottish Intercollegiate Guidelines Network. To assess selection bias, it was determined if an adequate concealment method was used. Centralised allocation, computerised allocation systems, and the use of opaque envelopes were regarded as adequate methods of concealment. To assess performance/detection bias, it was determined if the study was blinded. Blinding of the participants and therapists is difficult to ensure in cognitive treatment trials. Therefore, it was only appraised if the assessment of the outcome measures was conducted by a blinded person. To assess reporting bias, it was determined if the data was adequately reported.

### Statistical Analysis

All analyses were performed using Biostat software “Comprehensive Meta Analysis Version 2.2.064” [20]. Because of substantial differences between the treatment approaches that were evaluated in the included studies, there is no reason to assume that all studies share an identical true effect size. Consequently, a random effects model was used for the meta-analysis.

## Results

Of the randomized-controlled trials that were identified by the literature search, only 22 met all inclusion criteria and could be included in the meta-analysis. Interrater-agreement for article inclusion or exclusion exceeded κ = 0.786. All discrepancies were resolved by discussion. Coding reliabilities (percentage of interrater-agreement) for study characteristics and data extraction averaged 87%. Again, all discrepancies were disputed and solved.

Specifications regarding the methodological quality of the included trials were often incomplete. A sufficient description of the allocation concealment was missing in each of the 22 trials. Sixteen trials did not specify if the dependent variables were assessed by a blinded person [21]–[36]. Two trials [37], [38] stated explicitly that the outcome measures were assessed by a person that was aware of the study subjects’ affiliation. Four studies [39]–[42] performed a blind assessment of treatment outcomes. It can therefore be concluded that most studies are at risk of having a bias. Data was considered as adequately reported in all of the included trials. One trial had to be excluded from the analysis due to lack of information regarding outcome data. Attempts to contact the authors failed.

**Figure 2:**
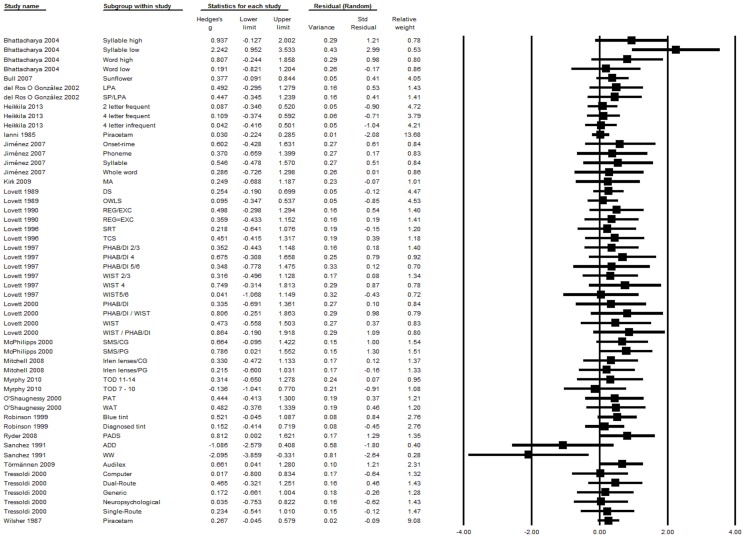
Treatment efficacy on reading performance. Funnel plot displays treatment efficacy on reading performance for each comparison of an experimental group with a control group. ADD = Adding phonemes; CG = Control group; DI = Direct instruction; DS = Decoding skills; EXC = Exceptional; LPA = Sound-symbol correspondence and phonemic awareness; MA = Morphological awareness; OWLS = Oral and written language skills; PADS = Phonemic awareness and decoding skills; PAT = Phonological awareness training; PG = Placebo-control group; PHAB = Phonological analysis and blending; REG = Regular; SMS = Specific motor sequence; SP = Speech perception; SRT = Strategy reciprocal teaching; TCS = Text content and structure; TOD = Temporal order detection; WIST = Word identification strategy training; WAT = Word analogy training; WW = Write a word.

**Figure 3:**
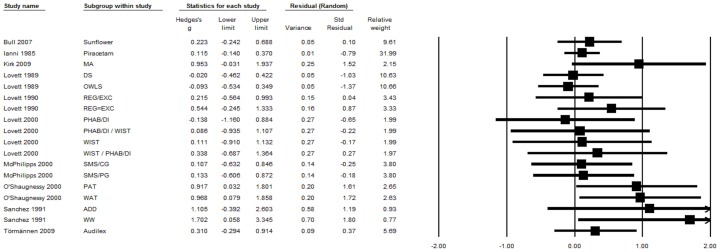
Treatment efficacy on spelling performance. Funnel plot displays treatment efficacy on spelling performance for each comparison of an experimental group with a control group. ADD = Adding phonemes; CG = Control group; DI = Direct instruction; DS = Decoding skills; EXC = Exceptional; MA = Morphological awareness; OWLS = Oral and written language skills; PAT = Phonological awareness training; PG = Placebo-control group; PHAB = Phonological analysis and blending; REG = Regular; SMS = Specific motor sequence; WIST = Word identification strategy training; WAT = Word analogy training; WW = Write a word.

Table S1 presents an overview of the trials that are included in the meta-analysis. Thirteen of the 22 trials included more than one intervention group, and two trials included more than one control group. Therefore, the meta-analysis was computed with a total of 49 comparisons of an experimental and a control group. These comparisons comprised 1138 participants in the experimental groups and 764 participants in the control groups. Effect size data for each subgroup within a study is presented separately for reading and spelling performance in Figure 2 and Figure 3.

The comparisons were distributed across the treatment approaches as follows: five reading fluency trainings, three phonemic awareness instructions, three reading comprehension trainings, 29 phonics instructions, three auditory trainings, two medical treatments, and four coloured overlays or lenses. One trial evaluated the effectiveness of sunflower therapy and another investigated the effectiveness of specific motor sequences. These two interventions could not be allocated to a category because they pursue an entirely different treatment approach. Results of the meta-analysis are reported separately for reading and spelling performance.

### Reading Performance

All included studies reported the results of reading measures, which made it possible to estimate each intervention’s efficacy regarding reading performance. Phonics instruction was investigated most often. This approach is the only one whose effectiveness on reading performance was statistically confirmed. The mean effect size for phonics instruction was *g*’ = 0.322 (95% CI [0.177, 0.467]; *I* ^2^ = 0). This suggests a small but statistically significant effect of phonics instructions on reading performance. The *I* ^2^ statistic describes the proportion of observed dispersion that reflects real differences rather than differences that occur by chance. As can be seen in Table 1, the mean effect sizes of the remaining treatment approaches did not reach statistical significance. Subgroup analysis revealed no statistically significant difference between treatment approaches (*p = *.788).

In addition, subgroup analyses were conducted to explore the influence of other variables (intervention and sample characteristics) on reading improvement. Results are displayed in Table 2. Studies that did not include or did not specify a certain variable were excluded from the subgroup analysis in question. In addition, it was not possible to define subgroups of age or grade level because children’s age and grade level showed considerable overlap between studies. Therefore, it was not possible to perform subgroup analyses with these variables.

**Table 2:** Subgroup analyses to explore the influence of variables on reading performance.

				95% CI		Heterogeneity				Significance		
Variable	Value	N	*g’*	Lower	Upper	*Q*	df	*p*	*I^2^*	*Q*	df	*p*
Severity	Mild reading disability	20	0.449	0.239	0.659	2.893	19	1.000	0%	3.339	2	0.188
	Moderate reading disability	23	0.228	0.113	0.342	32.037	22	0.077	31%			
	Severe reading disability	9	0.305	0.033	0.576	4.508	8	0.000	0%			
Amount	Up to 14 hours	17	0.351	0.181	0.520	16.023	16	0.450	0%	2.774	2	0.250
	Between 15 hours and 34 hours	12	0.113	−0.148	0.374	10.650	11	0.473	0%			
	35 hours and more	15	0.371	0.172	0.570	5.747	14	0.972	0%			
Duration	Up to 12 weeks	35	0.261	0.155	0.368	23.927	34	0.901	0%	0,618	1	0.432
	More than 12 weeks	17	0.353	0.151	0.554	18.231	16	0.311	12%			
Setting	Computer with teacher	9	0.364	0.085	0.643	2.766	8	0.948	0%	1.818	2	0.403
	Single subject	11	0.205	0.003	0.407	23.503	10	0.009	57%			
	Group	22	0.379	0.211	0.549	6.173;	21	0.999	0%			
Conductor	Study autor	5	0.806	0.397	1.215	6.446	4	0.168	38%	6.543	3	0.088
	Student	3	0.400	−0.109	0.909	0.144	2	0.931	0%			
	Teacher	13	0.247	0.046	0.449	6.046	12	0.914	0%			
	Special education therapist	20	0.256	0.090	0.422	13.622	19	0.805	0%			
Spelling/writing	Included	6	0.152	−0.157	0.451	7.332	5	0.197	32%	1.137	1	0.286
	Not included	34	0.331	0.195	0.467	24.473	33	0.858	0%			

The analyses revealed that intervention studies with mild reading disabled children and adolescents report a slightly higher mean effect size (*g’* = 0.449; 95% CI [0.239, 0.659]; *I* ^2^ = 0%) compared with studies that included moderately disabled (*g*’ = 0.228; 95% CI [0.113, 0.342]; *I* ^2^ = 31%) or severe reading disabled (*g’* = 0.305; 95% CI [0.033, 0.576]; *I* ^2^ = 0%) study subjects. However, this difference did not reach statistical significance (*p* = .188).

Studies were allocated into three distinct subgroups depending on the amount of intervention that was provided. No significant difference (*p* = .250) was found between the mean effect size of interventions that lasted up to 14 hours (*g’* = 0.351; 95% CI [0.181, 0.520]; *I* ^2^ = 0%), interventions that lasted between 15 hours and 34 hours (*g’* = 0.113; 95% CI [−0.148, 0.374]; *I* ^2^ = 0%), and interventions with more than 35 hours (*g’* = 0.371; 95% CI [0.172, 0.570]; *I* ^2^ = 0%).

To compare the effects of interventions with short- and long-term duration, the studies were divided into two subgroups: (a) up to 12 weeks; and (b) more than 12 weeks. The cut-off value of 12 weeks was chosen because it results in two subgroups of equal size making a statistical comparison between the two groups more appropriate. Interventions with a maximum duration of 12 weeks showed a small mean effect size of *g’* = 0.261 (95% CI [0.155, 0.368]; *I* ^2^ = 0%). Interventions that lasted more than 12 weeks tended to show higher effect sizes (*g’* = 0.353; [0.151, 0.554]; *I* ^2^ = 12%). Again, this difference did not reach statistical significance (*p* = .432).

To detect the impact of the setting on the success of an intervention three subgroups could be differentiated: (a) computer with teacher; (b) individual intervention; and (c) group intervention. The mean effect sizes of these subgroups did not differ significantly from each other (*p* = .403). The studies in the computer with teacher subgroup reached a mean effect size of *g’* = 0.364 (95% CI [0.085, 0.643]; *I* ^2^ = 0%), which was comparable to the mean effect size of group interventions (*g’* = 0.379; 95% CI [0.211, 0.549]; *I* ^2^ = 0%). Single subject interventions showed a small but significant mean effect size of *g’* = 0.205 (95% CI [0.003, 0.407]; *I* ^2^ = 57%).

Interventions that were conducted by the study author showed a high mean effect size (*g’* = 0.806; 95% CI [0.397, 1.215]; *I* ^2^ = 38%), whereas interventions that were conducted by teachers (*g’* = 0.247; 95% CI [0.046, 0.449]; *I* ^2^ = 0%) or special education therapists (*g’* = 0.256; 95% CI [0.090, 0.422]; *I* ^2^ = 0%) led to negligible mean effect sizes. Interventions that were conducted by students reached a small mean effect size (*g’* = 0.400; [−0.109, 0.909]; *I* ^2^ = 0%). Although a trend could be identified, there was no significant difference between these subgroups (*p* = .088).

In addition, subgroup analysis showed that the mean effect size of studies that did not include spelling/writing activities is moderate and significantly greater than zero (*g’* = 0.331; 95% CI [0.195, 0.467]; *I* ^2^ = 0%). Interventions that included spelling/writing exercises showed a small effect on reading improvement that did not reach statistical significance (*g’* = 0.152; 95% CI [−0.157, 0.451]; *I* ^2^ = 32%). This difference did not reach statistical significance (*p* = .286).

### Spelling Performance

Ten trials (containing 18 comparisons) conducted spelling tests before and after treatment. It was, therefore, possible to calculate 18 effect sizes for spelling. Only in case of phonics instruction was it possible to compute a mean effect size. The other treatment approach categories included only one study that assessed spelling performance. Ten studies evaluated the effect of phonics instruction on spelling performance. These revealed a small but statistically significant mean effect size (*g’* = 0.336; 95% CI [0.062, 0.610]; *I* ^2^ = 22%).

Again, subgroup analyses were conducted to explore the involvement of other variables (intervention and sample characteristics) on the improvement of spelling performance. Because only few studies were available, some subgroups comprised less categories as in the case of reading performance (see Table 3).

**Table 3:** Subgroup analyses to explore the influence of variables on spelling performance.

				95% CI		Heterogeneity				Significance		
Variable	Value	N	*g’*	Lower	Upper	*Q*	df	*p*	*I^2^*	*Q*	df	*p*
Severity	Mild reading disability	8	0.415	0.089	0.741	4.965	7	0.664	0%	1.830	1	0.176
	Moderate reading disability	8	0.157	−0.027	0.340	9.712	7	0.205	28%			
Amount	Up to 14 hours	4	0.432	0.114	0.749	3.481	3	0.323	14%	9.295	2	0.010
	Between 15 hours and 34 hours	3	1.140	0.404	1.875	0.589	2	0.745	0%			
	35 hours and more	8	0.059	−0.181	0.300	2.620	7	0.918	0%			
Duration	Up to 12 weeks	9	0.176	0.011	0.341	9.209	8	0.325	13%	0.542	1	0.462
	More than 12 weeks	9	0.314	−0.015	0.643	7.061	8	0.530	0%			
Setting	Single subject	3	0.488	−0.061	1.038	3.817	2	0.148	48%	0.509	1	0.476
	Group	11	0.266	0.000	0.532	11.565	10	0.315	14%			
Conductor	Student	3	0.945	0.417	1.474	0.007	2	0.997	0%	7.734	2	0.021
	Teacher	4	0.099	−0.412	0.610	0.417	3	0.937	0%			
	Special education therapist	7	0.148	−0.082	0.378	7.793	6	0.254	23%			
Spelling/writing	Included	5	0.371	−0.067–	0.809	7.814	4	0.099	49%	0.013	1	0.908
	Not included	8	0.337	−0.038	0.713	8.111	7	0.323	14%			

Studies with participants considered as mild reading disabled (*g’* = 0.415; 95% CI [0.089, 0.741]; *I* ^2^ = 0%) showed a statistically significant mean effect size on spelling performance, whereas the effectiveness of studies with moderately disabled study subjects (*g’* = 0.157; 95% CI [−0.027, 0.340]; *I* ^2^ = 28%) could not be statistically confirmed. However, the analysis revealed no statistically significant difference between these two categories of severity (*p* = .176).

Significant differences (*p* = .010) were found between the mean effect sizes of interventions that lasted up to 14 hours (*g’* = 0.432; 95% CI [0.114, 0.749]; *I* ^2^ = 14%), interventions that lasted between 15 hours and 34 hours (*g*’ = 1.140; 95% CI [0.404, 1.875]; *I* ^2^ = 0%), and interventions with more than 35 hours (*g*’ = 0.059; 95% CI [−0.181, 0.300]; *I* ^2^ = 0%). In contrast, it was found that interventions that lasted more than 12 weeks have a higher mean effect size (*g*’ = 0.314; [−0.015, 0.643]; *I* ^2^ = 0%) than interventions with a maximum duration of 12 weeks (*g’* = 0.176; [0.011, 0.341]; *I* ^2^ = 13%). However, this difference failed to reach statistical significance (*p* = .462).

Interventions that were conducted by teachers (*g’* = 0.099; 95% CI [−0.412, 0.610]; *I* ^2^ = 0%) or special education therapists (*g’* = 0.148; 95% CI [−0.082, 0.378]; *I* ^2^ = 23%) led to negligible mean effect sizes. Interventions that were conducted by students reached a large mean effect size (*g’* = 0.945; 95% CI [0.417, 1.474]; *I* ^2^ = 0%). This difference reached statistical significance (*p* = .021).

The mean effect sizes of studies that investigated individually administered interventions and studies that investigated group interventions did not differ significantly from each other (*p* = .476). Single subject interventions showed a mean effect size of *g’* = 0.488, which was not statistically greater than zero (95% CI [−0.061, 1.038]; *I* ^2^ = 48%). Group interventions showed a mean effect size of *g’* = 0.266 (95% CI [0.000, 0.532]; *I* ^2^ = 14%).

The mean effect size of studies that did not include spelling/writing activities (*g’* = 0.337; 95% CI [−0.038, 0.713]; *I* ^2^ = 14%) did not significant differ (*p* = .908) from the mean effect size of interventions that included spelling/writing exercises (*g’* = 0.371; 95% CI [−0.067, 0.809]; *I* ^2^ = 49%).

### Additional Analyses

In the vast majority of studies (19 out of 22), the effect size calculation was based on standardized measures. Only three trials [23], [26], [30] used non-standardized measures of learning transfer. These studies had evaluated phonics instructions, reading comprehension trainings, and a reading fluency training. Because the inclusion of studies with non-standardized measures might introduce an artifact (outlined above), the main analyses were rerun after these three studies were excluded.

Since only one study remained in the category ‘reading comprehension training’, it was not possible to calculate a mean effect size for this treatment approach. In the category ‘reading fluency training’ the exclusion of studies with non-standardized measures led to a minor change in the magnitude of the effect (Reading: *g*’ = 0.280; 95% CI [−0.072, 0.322]); *n* = 4). Interestingly, the mean effect sizes for phonics instruction are higher if trials using non-standardized measures are excluded from the analysis (Reading: *g*’ = 0.424; 95% CI [0.246, 0.601]; *n* = 25; Spelling: *g*’ = 0.376; 95% CI [0.065, 0.686]); *n* = 9). These findings demonstrate that the inclusion of studies with non-standardized measures in the present meta-analysis did not lead to an overestimation of the effect sizes and, therefore, does not confound the results.

### Publication Bias

A common problem of all disciplines in meta-analytic reviews is the phenomenon of publication bias [43]. Publication bias occurs because statistically significant results are more likely to be published than non-significant results.

**Figure 4:**
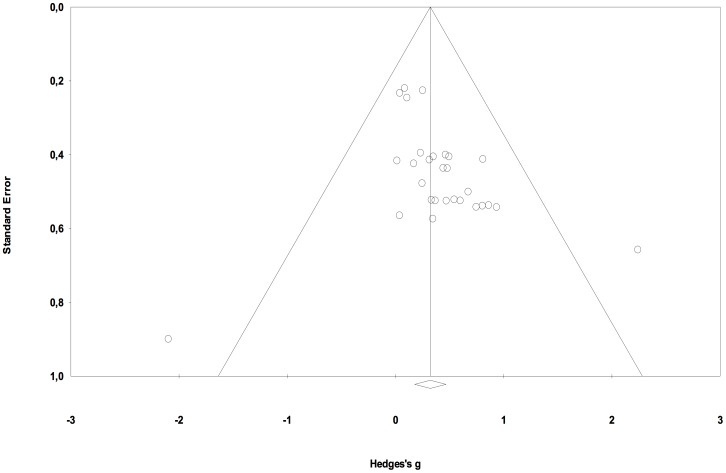
Funnel plot of standard error by Hedges *g* for observed comparisons. Funnel plot displays observed comparisons evaluating the efficacy of phonics instructions on reading performance.

**Figure 5:**
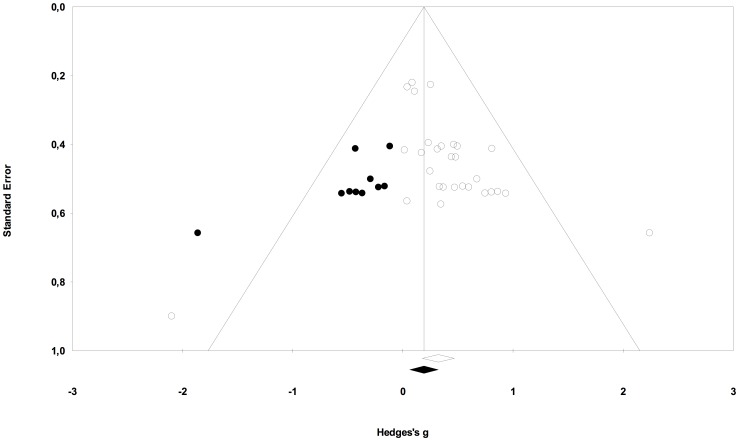
Funnel plot of standard error by Hedges *g* for observed and imputed comparisons. Funnel plot displays observed and imputed comparisons evaluating the efficacy of phonics instructions on reading performance.

Only a small number of included studies assessed spelling performance. In addition, phonics instruction is the only treatment approach whose positive effect on reading performance is statistically confirmed. Therefore, publication bias was explored exemplarily only for those studies that evaluated phonics instruction and used reading performance as dependent variable. A funnel plot was used to explore the presence of publication bias. The shape of the funnel plot displayed asymmetry with a gap on the left of the graph. Using Duval and Tweedie’s trim and fill [44] the extent of publication bias was assessed and an unbiased effect size was estimated. This procedure trimmed 10 studies into the plot and led to an estimated unbiased effect size of *g’* = 0.198 (95% CI [0.039, 0.357]) (see Figures 4 and 5, Table 4).

**Table 4:** Unbiased effect size estimation for the efficacy of phonics instruction on reading performance.

			95% CI		
	Studies trimmed	*g*	Lower	Upper	*Q*
Observed		0.322	0.177	0.467	26.810
Adjusted	10	0.198	0.039	0.357	50.228

## Discussion

The first aim of this meta-analysis was to determine the effectiveness of different treatment approaches on reading and spelling performance of reading disabled children and adolescents. The results reveal that phonics instruction is the most intensively investigated treatment approach. In addition, it is the only approach whose effectiveness on reading and spelling performance in children and adolescents with reading disabilities is statistically confirmed. This finding is consistent with those reported in previous meta-analyses [9], [45]. At the current state of knowledge, it is adequate to conclude that the systematic instruction of letter-sound-correspondences and decoding strategies, and the application of these skills in reading and writing activities, is the most effective method for improving literacy skills of children and adolescents with reading disabilities. The treatment approach phonics instruction has not only been evaluated in English-speaking countries, but also in studies conducted in Spain, Finland, and Italy. Despite the widespread use of this approach, it is not yet clear whether these interventions are equally effective across languages. This question could not be addressed in the present analysis and needs to be addressed by further research.

Phonics instruction combines elements of reading fluency training and phonemic awareness training. Reading fluency trainings emphasize repeated word or text reading practice. The results of the present meta-analysis suggest that reading fluency training alone is not an effective way to enhance the reading and spelling skills of children and adolescents with reading disabilities, as was reported in a previous meta-analysis [14].

Phonemic awareness trainings are widely recognised as being effective for the remediation of preschool children at risk for reading disabilities [46], [47]. The present results demonstrate that when phonemic awareness interventions are provided to school-aged children and adolescents with reading difficulties, they do not have a significant effect on a child’s reading or spelling performance. This indicates that phonemic awareness and reading fluency trainings alone are not sufficient to achieve substantial improvements. However, the combination of these two treatment approaches, represented by phonics instruction, has the potential to increase the reading and spelling performance of children and adolescents with reading disabilities.

In terms of reading comprehension training, it was not possible to confirm a significant influence of this approach on literacy achievement. This result should be interpreted with caution because the present meta-analysis included only three comparisons that evaluated reading comprehension training. All three comparisons were conducted by the same author and they demonstrated negligible [28] to small [26] effect sizes. There is a clear need to complement these studies with further research.

The mean effect size of coloured lenses (Irlen lenses) did not reach statistical significance. Some studies compared the effect of coloured lenses to a placebo control group; other studies used an untrained control group instead. An interesting observation is that Irlen lenses showed small effect sizes if the experimental group was compared to an untreated control group [41]. If the experimental group was compared to a placebo control group, effect sizes were negligible [33], [41]. This finding confirms earlier systematic reviews that could not prove any positive effect of coloured lenses on literacy achievement, and suggests that results are mainly due to placebo effects [48], [49].

Studies that tried to enhance reading and spelling skills of children and adolescents with reading disabilities by medication with the nootropic piracetam showed only minor effects, and the mean effect size for reading performance did not reach statistical significance. With the possibility of side effects in mind [50] the risks of medication seem to outweigh its benefits.

Auditory trainings intend to foster reading and spelling by focussing on the underlying causes of the poor performance. At first glance, this approach seems convenient, but the results of the present meta-analysis demonstrate that auditory trainings do not significantly improve children’s reading and spelling skills. Based on the results of the present meta-analysis and those reported by other systematic reviews and non-randomized trials [10], [51], [52], it can be concluded that focussing directly on literacy skills is effective but the efficacy of interventions focussing on the underlying causes could not be confirmed to date.

The second aim of this meta-analysis was to explore the impact of various factors on the efficacy of interventions. The results of subgroup analyses do not allow clear conclusions about what makes an intervention successful. This may be caused by mutual confounding in the subgroup analyses, which means that each moderator could be confounded by any of the other moderators. This influences the observed association between moderator and outcomes and distorts the true magnitude of effects. As a consequence, the results of the performed subgroup analyses should be interpreted with caution. However, some findings are worth noting. First, subgroup analyses demonstrated that children and adolescents with mild reading disabilities show more improvement in literacy skills than more severely impaired participants. Second, interventions with higher amounts of treatment or longer durations of treatment seem to be more effective in improving literacy skills than therapies with small amounts of treatment or short-time interventions. Third, consistent with previous meta-analyses [8], [14], it was found that interventions that were conducted by the study author tend to show higher effect sizes than interventions that were implemented by other conductors. This suggests that solid and professional knowledge about reading disability in children and adolescents might enhance treatment efficacy. Meta-regression or hierarchical linear methods can be helpful to identify specific variables that influence the efficacy of an intervention. Due to the small number of included studies that distinguished or evaluated each variable, these statistical methods could not be applied in the present meta-analysis.

Unfortunately, it could not be assessed which intervention is particularly effective for a specific age or grade level. This was due to the occurrence that many of the included trials comprised study subjects of a wide age span. Ever since the meta-analyses of the NRP in the year 2000 [8], it has been apparent that interventions are not equally effective for different age groups or grade levels. Providing children of a wide age span with the same interventions is therefore not a recommended option for research settings and clinical practice.

The influence of publication bias was determined with funnel plots. Publication bias refers to the appearance that many studies remain unpublished because of negligible effect sizes or non-significant findings [53]. This is presumably the case in this research domain. We controlled publication bias exemplarily for the treatment approach of phonics instructions, but it can be assumed that this phenomenon is present in the other treatment approaches as well. Duval and Tweedies trim and fill analysis estimated and valued the true, unbiased effect size as being small, but still statistically significant.

Consistent with prior research [9], [11], [12], [14], [45], this analysis demonstrated that severe reading and spelling difficulties can be ameliorated with appropriate treatment. The need for evidence-based interventions is obvious given the emotional and academic consequences for children with persistent reading disorders [6]. To increase the informative value of studies, research in this domain should improve its methodological quality. Studies were often excluded from this analysis because of the absence of randomized allocation concealment. Randomization tries to secure that known and unknown determining factors are spread equally across groups. Research has shown that when meta-analyses include studies whose allocation concealment is inadequate, effects of interventions can be misjudged [54]. Each study that was included in our analysis was randomized, but due to missing methodological specifications the quality of randomization procedures could not be determined. An equally important aspect is the assessment of the dependent variables by a blinded person. It has been demonstrated [55], [56] that effects of interventions are exaggerated if the relevant outcome measures are not assessed in a blinded test situation. Therefore, effects can only be attributed to the conducted intervention if they are observed in a blinded randomized controlled trial with an adequate concealment technique. Unfortunately, most of the studies included in the present meta-analysis did not specify whether the dependent variable was assessed by a blinded person.

This meta-analysis comprises studies from various English-speaking and non-English-speaking countries like Finland, Italy, Spain, and Brazil. To conduct a meaningful meta-analysis with an adequate number of comparisons, these studies could not be analyzed separately for different languages or groups of languages. The transferability of research findings from English-speaking countries to languages with more consistent orthographies and less syllabic complexity and vice versa is largely debated [57]–[59]. It has been demonstrated that differences between languages affect children’s literacy acquisition [59], [60] and, therefore, it cannot be generally assumed that symptom based treatment approaches are equally effective in each language.

The Anglo-American region far outweighs other countries in quantity and quality of the published work in this research domain. In order to be able to support children and adolescent with reading disabilities in different languages with evidence-based interventions, research in every country has to realign on high-quality standards. This refers in particular to the intensified application of blinded randomized controlled trials. Moreover, in order to solve the questions of the transferability of research findings across languages, cross-linguistic studies are required.

## Supporting Information

Table S1 **Study characteristics.**
(DOCX)

Checklist S1 **PRISMA Checklist.**
(DOC)
