# Correction: Effectiveness of Treatment Approaches for Children and Adolescents with Reading Disabilities: A Meta-Analysis of Randomized Controlled Trials

**DOI:** 10.1371/journal.pone.0105843

**Published:** 2014-08-12

**Authors:** The PLOS ONE Staff

In the Methods section, there are errors in Formula 1- Hedges *g*. The term (n_EG_ -1)s^2^_CG_ appears under the root; the correct term is (n_CG_ -1)s^2^_CG_. In addition, the parentheses for the correction factor are missing. Please view the complete, correct formula here:



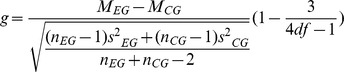



## References

[1] Galuschka K, Ise E, Krick K, Schulte-Körne G (2014) Effectiveness of Treatment Approaches for Children and Adolescents with Reading Disabilities: A Meta-Analysis of Randomized Controlled Trials. PLoS ONE 9(2): e89900 doi:10.1371/journal.pone.0089900 24587110 10.1371/journal.pone.0089900 PMC3935956

