# Supplementary material for: Effectiveness of Treatment Approaches for Children and Adolescents with Reading Disabilities: A Meta-Analysis of Randomized Controlled Trials
Source: PLoS One. 2014 Feb 26;9(2):e89900. doi: 10.1371/journal.pone.0089900 (PMC3935956; doi:10.1371/journal.pone.0089900)
Supplement: Table S1 — Study characteristics. (DOCX) [file pone.0089900.s001.docx]

**Table S1. Study characteristics.**

| **First author and year of publication** | **Approach** | **Experimental group** | **Content** | **Control condition** | **Amount** | **Setting** | **Conductor** | **Inclusion criteria: reading** | **Age/Grade** |
| --- | --- | --- | --- | --- | --- | --- | --- | --- | --- |
| Bhattacharya 2004 [37] | Phonics instruction | Syllable low | Children with a reading score on third grade level practiced reading of a total of 100 words (25 per session). The words were orally divided in syllables with supporting hand signals. | No- treatment control group | Four days within a week, one session lasted 30 minutes.  Total amount: 2 hours | Single subject | Research team | ≥ 1 year below age norm | Age: range not specified  Grade: 6 to 10 |
|  | Phonics instruction | Syllable high | Children with a reading score on fourth or fifth grade level practiced reading of a total of 100 words (25 per session). The words were orally divided in syllables with supporting hand signals. |  |  |  |  |  |  |
|  | Reading fluency training | Word low | Children with a reading score on third grade level practiced reading of a total of 100 words (25 per session) by reading them as whole words rather than in parts. |  |  |  |  |  |  |
|  | Reading fluency training | Word high | Children with a reading score on fourth or fifth grade level practiced reading of a total of 100 words (25 per session) by reading them as whole words rather than in parts. |  |  |  |  |  |  |
| Bull 2007 [21] | No category | Sunflower | The therapy combined homeopathy, acupressure, osteopathy and kinesiology to address the study subjects’ personal, neurological and motivational deficits. | No- treatment control group | Eight appointments, one session lasted 40 minutes.  Total amount: 5:20 hours | Single subject | Sunflower practitioner | Diagnosed dyslexia by an educational psychologist | Age: 6 to 13 years  Grade: not specified |
| del Rosario Ortiz Gonzáles 2002 [22] | Phonological awareness training | Speech perception, sound-symbol correspondence and phonemic awareness  (SP/LPA) | The program contained a systematic instruction in letter-sound correspondence and phonemic awareness as well as speech perception. All tasks were presented and performed orally. | No- treatment control group | Four weeks, five times a week, one session lasted 20 minutes.  Total amount: 6:30 hours | Groups with 4 subjects | Postgraduate student | ≤ 15 percentile | Age: 9;0 to 11;0 years  Grade: not specified |
|  | Phonological awareness training | Sound-symbol correspondence and phonemic awareness (LPA) | The program contained a systematic instruction in letter-sound correspondence and phonemic awareness. All tasks were presented and performed orally. |  |  |  |  |  |  |
| Heikkila 2013 [23] | Phonics instruction | 2-letter frequent | Study subjects practiced 30 2-letter frequent syllables, and repeated each of them 50 times. During training, participants heard an auditory stimulus via headphones and chose the corresponding syllable as quickly as possible from five written options on the computer screen. They received feedback according to the speed of accurate responses. | Placebo control group | Ten sessions in two or three weeks, one session lasted five to ten minutes.  Total amount: ca. 1:30 hours | Single subject | Teacher | ≥ 1 SD below expected level | Age: 8;3 to 11;3  Grade: not specified |
|  | Phonics instruction | 4-letter frequent | Study subjects practiced 30 4-letter frequent syllables, and repeated each of them 50 times. During training, participants heard an auditory stimulus via headphones and chose the corresponding syllable as quickly as possible from five written options on the computer screen. They received feedback according to the speed of accurate responses. |  |  |  |  |  |  |
|  | Phonics instruction | 4-letter infrequent | Study subjects practiced 30 4-letter infrequent syllables, and repeated each of them 50 times. During training, participants heard an auditory stimulus via headphones and chose the corresponding syllable as quickly as possible from five written options on the computer screen. They received feedback according to the speed of accurate responses. |  |  |  |  |  |  |
| DiIanni 1985 [39] | Medical Treatment | Piracetam | The subjects received a daily dose of 3.3g Piracetam | Placebo control group | Twelve weeks; 3.3 g per day | - | - | RQ ≤ .85 | Age: 8;0 to 13;11  Grade: not specified |
| Jimenéz 2007 [24] | Phonics instruction | Phoneme | Words were presented on a computer screen. Children read these words phoneme by phoneme. | No- treatment control group | Three weeks, five times a week, one session lasted 30 to 40 minutes.  Total amount: 8:45 hours. | Single subject | Psychologist | < 25 percentile | Age: 7;1 to 10;6  Grade: not specified |
|  | Phonics instruction | Syllable | Words were presented on a computer screen. Children read these words syllable by syllable. |  |  |  |  |  |  |
|  | Reading fluency training | Whole-word | Words were presented on a computer screen. Children read these words as a whole. |  |  |  |  |  |  |
|  | Phonics instruction | Onset-rime | Words were presented on a computer screen. Children read onset and rime segments in monosyllabic words. |  |  |  |  |  |  |
| Kirk 2009 [25] | Phonics instruction | Morphological awareness  (MA) | The subjects trained the identification of vowel length and orthographic rules. The program provided an insight to morphology and included spelling training. | No- treatment control group | Three months, two times a week, one session lasted 45 minutes.  Total amount: ca. 19:30 hours | Half as single subject, half in groups (2 subjects) | First author, speech-language pathology students | ≥ 1 SD below expected age norm | Age: 8;7 to 11;1 years  Grade: not specified |
| Lovett 1989 [28] | Reading comprehension | Oral and written language skills  (OWLS) | The program included speech comprehension exercises, reading and reading comprehension tasks as well as spelling. | Placebo control group | 4 times a week, 50- 60 minutes. The training lasted for 10 weeks.  Total amount: 33 – 40 hours | Groups (2 subjects) | Special education teacher | ≥ 1.5 SD below expected age norm | Age: 8 to 13 years  Grade: not specified |
|  | Phonics instruction | Decoding skills program  (DS) | The program contained the training of grapheme-phoneme correspondence rules, written spelling and phoneme analysis and blending. |  |  |  |  |  |  |
| Lovett 1990 [30] | Phonics instruction | Regular not like exceptional  (REG/EXC) | The program contained systematic instruction of word recognition and spelling skills. Regular words were taught by training the consistent letter sound mappings. Exception words were rehearsed by whole-word methods. | Placebo control group | 9 weeks 35 hours 4 times a week 60 minutes. The training lasted 9 weeks  Total amount: 35 hours training | Groups  (subjects) | Special education teacher | < 25 percentile | Age: 7 to 13 years  Grade: not specified |
|  | Reading fluency training | Regular like exceptional  (REG=EXC) | In this treatment, both regular and exception words were taught by the whole-word method. |  |  |  |  |  |  |
| Lovett 1996 [26] | Reading comprehension training | Text content and structure  (TCS) | Subjects learned to extract textual information and to relate it to existing knowledge. | Placebo control group | 4 days a week 60 minutes training. The intervention lasted 6 to 7 weeks.  Total amount: 25 hours of training | Groups (2 subjects) | Special education teacher | < 25 percentile | Age: range not specified  Grade: 7 to 8 |
|  | Reading comprehension training | Strategy reciprocal teaching  (SRT) | Subjects learned text comprehension strategies. Four operations were instructed: summarizing, questioning, clarifying and predicting. |  |  |  |  |  |  |
| Lovett 1997 [29] | Phonics instruction | Phonological analysis and blending, direct instruction with children on second and third grade level  (PHAB/DI 2/3) | The program contained exercises for phonological analysis and blending. Materials were presented orally and printed as texts. | Placebo control group | Eight or nine weeks, 4 times a week, each session lasted 60 minutes.  Total amount: 35 hours. | Groups (2-3 subjects) | Teacher | < 20 percentile | Age: 7 to 12 years  Grade: 2 to 6 |
|  |  | Phonological analysis and blending, direct instruction with children on fourth grade level  (PHAB/DI 4) |  |  |  |  |  |  |  |
|  |  | Phonological analysis and blending, direct instruction with children on fifth and sixth grade level  (PHAB/DI 5/6) |  |  |  |  |  |  |  |
|  | Phonics instruction | Word identification strategy training with children on second and third grade level  (WIST 2/3) | The program instructed children in the acquirement and use of word identification strategies. |  |  |  |  |  |  |
|  |  | Word identification strategy training with children on fourth grade level  (WIST 4) |  |  |  |  |  |  |  |
|  |  | Word identification strategy training with children on fifth and sixth grade level  (WIST 5/6) |  |  |  |  |  |  |  |
| Lovett 2000 [27] | Phonics instruction | Phonological analysis and blending, direct instruction (PHAB/DI ) | The program contained exercises for phonological analysis and blending. Materials were presented orally and printed as texts. | Placebo control group | 1 hour a day up to a total amount of  70 hours treatment. | Groups (3 subjects) | Teacher | < 20 percentile | Age: 6;9 to 13;9 years  Grade: not specified |
|  | Phonics instruction | Word identification strategy training  (WIST) | The program instructed children in the acquirement and use of word identification strategies. |  |  |  |  |  |  |
|  | Phonics instruction | Word identification strategy training before phonological analysis and blending, direct instruction  (WIST/ PHAB /DI) | In the first 35 sessions WIST was conducted, in the last 35 hours, the PHAB/DI program was used. |  |  |  |  |  |  |
|  | Phonics instruction | Phonological analysis and blending, direct instruction before word identification strategy training  (PHAB/DI/ WIST) | In the first 35 sessions PHAB/DI was conducted, in the last 35 hours, the WIST program was used. |  |  |  |  |  |  |
| McPhillips 2000 [42] | No category | Specific movement sequence  (SMS) | The study subjects performed some special motor exercises to inhibit the asymmetric tonic neck reflex (ATNR). | No-treatment control group and Placebo control group | 52 weeks | Single subject | - | ≥2 years below age norm | Age: 8 to 11 years  Grade: not specified |
| Mitchell 2008 [41] | Colored overlays | Irlen lenses | Everyday reading activities were done with Irlen-lenses. | No-treatment control group and Placebo Control group | 4 weeks | - | - | Diagnosed dyslexia | Age: 7;11 to 11; 9  Grade: not specified |
| Murphy 2010 [31] | Auditory training | Temporal order detection with 7 to 10 years old participants  (TOD 7 - 10) | The training contained auditory stimuli with multiple frequencies. The study subjects classified them as ascending and descending. | No-treatment control group | 5 sessions per week with 20 minutes in 9 weeks  Total amount: 15 hours training | Single subject | - | 2 years below expected age norm | Age: 7 to 14 years  Grade: not specified |
|  |  | Temporal order detection 11 to 14 years old participants  (TOD 11 - 14) |  |  |  |  |  |  |  |
| O’Shau-ghnessy 2000 [32] | Phonics instruction | Phonological awareness training (PAT) | The intervention contained elements of phonological awareness training (rhyming, sound blending and sound segmenting) as well as reading and spelling training directed at the level of phonemes. | Placebo control group | 6 weeks 3 times a week for 30 minutes. Total amount: 9 hours | Groups (5 subjects) | Para-professional | < 25 percentile | Age: range not specified  Grade: 2 |
|  | Phonics instruction | Word analogy training  (WAT) | The intervention contained training of phonological awareness through contextualized written language activities as well as reading and spelling training directed at the level of onsets and rimes. |  |  |  |  |  |  |
| Robinson 1999 [33] | Colored overlays | Diagnosed tint | All reading activities were done with tints in the probands preferred color. | Placebo control group | 13 – 17 weeks | - | - | ≥ 1 year below expected age norm | Age: 9,2 to 13,1 years  Grade: not specified |
|  | Colored overlays | Blue tint | All reading activities were done with blue tints. |  |  |  |  |  |  |
| Ryder 2008 [34] | Phonics instruction | Phonemic awareness and phonemically based decoding skills program (PADS) | The intervention contained training of phonological awareness, letter-sound-correspondences and training of phonemically based decoding strategies through reading of phonetically controlled storybooks. | No-treatment control group | 24 weeks 4 times a week for 25 minutes.  Total amount: 40 hours | Groups (3 subjects) | Teacher aide | ≥ 1 SD below expected age norm | Age: 6 to 7 years  Grade: 2 to 3 |
| Sanchez 1991 [35] | Phonological awareness training | Adding phonemes  (ADD) | The training taught strategies for adding a phoneme to a sequence. | Placebo control group | 22 weeks 2 sessions a week over 30 minutes  Total amount of 22 hours training | Single subject | Not specified | ≥ 1,5 years below grade level | Age: range not specified  Grade: 2 to 3 |
|  | Phonics instruction | Write a word  (WW) | The training taught strategies for adding a phoneme to a sequence and includes the use of correspondence rules between phonemes and letters and trained the implementation of the new knowledge in spelling. |  |  |  |  |  |  |
| Törmänen 2009 [38] | Auditory training | Audilex | Subjects are trained to match visual and auditory patterns. | No-treatment control group | 2 sessions a week for 15 minutes in 8 weeks.  Total amount: 4 hours training | Single subject | Author | Diagnosed dyslexia | Age: 7;4 to 12;5 years |
| Tressoldi 2000 [36] | Reading fluency training | Neuropsychological | Each participant of this group read a book while wearing a headphone that provided feedback of the study subjects’ voice only to the right ear. The book was positioned in the right hemispace. The auditory feedback and the book position should stimulate the left hemisphere. | No-treatment control group | 2 times a week for about 25 – 30 minutes. Overall 25 training sessions at an average of 12.5 weeks.  Total amount: 25 to 37 hours | Single subject | Trained clinician | 2 SD below expected age norm | Age: range not specified  Grade: 3 to 8 |
|  | Phonics instruction | Dual-Route | Participants read words, homophones and nonwords on a computer screen. Time of response and accuracy were fed back to the subject to stress automaticity. |  |  |  |  |  |  |
|  | Phonics instruction | Single-Route | Participants read words on a computer screen. By pressing the space bar key, they could get help with segregating and recognizing the syllables within a word. |  |  |  |  |  |  |
|  | Phonics instruction | Computer | Participants detected a graphemic target (letters, syllables or words) on a computer screen. |  |  |  |  |  |  |
|  | Phonics training | Generic | Participants read syllables and words on paper, cut words in syllables and composed words using isolated syllables etc. |  |  |  |  |  |  |
| Wilsher 1987 [40] | Medical treatment | Piracetam | The subjects received a daily dose of 3.3g Piracetam | Placebo control group | 36 weeks | - | - | Reading quotient  ≤ .85 | Age: 7;6 to 12;11  Grade: not specified |
